# Are You Willing to Protect the Health of Older People? Intergenerational Contact and Ageism as Predictors of Attitudes toward the COVID-19 Vaccination Passport

**DOI:** 10.3390/ijerph191711061

**Published:** 2022-09-04

**Authors:** Emilio Paolo Visintin, Alessandra Tasso

**Affiliations:** Department of Humanities, University of Ferrara, Via Paradiso 12, 44121 Ferrara, Italy

**Keywords:** intergenerational contact, ageism, COVID-19 vaccination, COVID-19 vaccination passport

## Abstract

Since the beginning of the COVID-19 pandemic, the vulnerability of older people to COVID-19 has been stressed in political discourse and the mass media, with the call to protect older adults. Therefore, policies aimed at reducing the spread of coronavirus, such as the COVID-19 vaccination passport policy, might be perceived as policies aimed at preserving the health of older people, and negative attitudes toward older people (i.e., ageism) might underlie negative attitudes toward such policies. While intergenerational contact is one of the main antecedents of reduced ageism, the pandemic has forced people to separate, and direct intergenerational contact in particular might have been reduced, possibly being replaced by telephone and virtual contact. In a correlational study (*N* = 153 Italian university students) we found that quantity and quality of direct intergenerational contact diminished during the pandemic. Virtual intergenerational contact was unexpectedly less frequent than direct contact. Quality of direct contact before the pandemic was associated, over and above the effects of other contact forms under investigation, with reduced ageism, which was in turn associated with negative attitude toward the vaccination passport. Findings will be discussed focusing on the roles of intergenerational contact and ageism for public health.

## 1. Introduction

All around the world, the lives of people of any age have dramatically changed since the beginning of the COVID-19 pandemic. To reduce the spread of SARS-CoV-2 (from now on, coronavirus) people have been asked to follow behavioral guidelines (e.g., wearing face masks, washing hands frequently and using sanitizing gel) and to get vaccinated against COVID-19. While most of the people have implemented such preventive behaviors, not everyone did, and there have been also harsh resistance and protests against face masks and against vaccinations [1]. Among the most controversial preventive measures, several countries implemented COVID-19 vaccination passport policies, i.e., people were allowed to enter several public and private spaces (e.g., restaurants and bars, workplaces, schools and universities) only if they had a COVID-19 vaccination passport certifying a full cycle of vaccination or a very recent negative COVID-19 test or that they previously had COVID-19. While the vaccination passport aimed at explicitly reducing the spread of coronavirus and implicitly increasing vaccination rates, because people who went through a complete COVID-19 vaccination cycle are less likely to get infected and to transmit coronavirus [2,3], part of public opinion, of cultural elites, and of politicians were against vaccination obligations and the vaccination passport, considering them as limitations of individual freedom and intrusions of government in private health choices [1,4]. This research analyzed predictors of attitude toward the COVID-19 vaccination passport, focusing on intergenerational contact and ageism (i.e., age-based stereotyping, prejudice and discrimination), and more specifically on non-older people contact with and prejudice against older adults.

Indeed, political discourse and the mass media have emphasized the vulnerability to COVID-19 of several social groups such as people with low socio-economic status who might have limited access to high quality healthcare, or people with debilitating diseases or older people who are at high risk of severe complications if infected by COVID-19. Focusing on older adults, Swift and Chasteen [5] and Ayalon et al. [6] emphasized the rhetoric used by the mass media to describe older adults as a vulnerable and fragile group. Moreover, in political discourse both benevolent (i.e., patronizing attitudes toward older people seen as a homogeneous and vulnerable group [7]) and hostile (i.e., blatant and explicitly negative attitudes toward older people [7]) forms of ageism emerged. For example, in Italy, a country with a large share of older population where we conducted this research, to encourage physical distance and preventive behaviors during the first lockdown the former Italian Prime Minister Giuseppe Conte stated that “we should preserve […] especially the health of our grandparents”, with a narrative which can be interpreted as benevolent ageism. Hostile ageism emerged in political debate in Italy as well, with calls for restrictions exclusively for older people described as “not indispensable to the country’s productive effort” by the President of Liguria Region Giovanni Toti. The upsurge of different forms of ageism during the pandemic has also been witnessed by research analyzing tweets [8,9,10] and older people’s experiences [11]. 

The narrative emphasizing the vulnerability of older people with the call to protect older adults, besides possibly reflecting and spreading benevolent ageism [6,12], should also emphasize that preventive behaviors and policies aimed at reducing the spread of coronavirus might contribute to preserving the health of older people. Therefore, policies such as the COVID-19 vaccination passport policy, which aimed at reducing the spread of coronavirus, might be perceived as policies aimed at preserving the health of older people. Previous research suggested that ageism was negatively associated with the implementation of preventive behaviors aimed at reducing the spread of coronavirus [13,14,15] or with attitudes toward measures aimed at protecting the health of older people during the COVID-19 pandemic [16]. In this vein, we proposed that ageism might be associated with negative attitude toward the COVID-19 vaccination passport policy.

While intergenerational contact is one of the main antecedents of reduced ageism [17,18,19], the pandemic has forced people to separate, and especially direct, face-to-face intergenerational contact might have been reduced since the beginning of the pandemic to preserve the health of older people [20]. However, in line with calls for intergenerational solidarity and not to leave older people alone during the pandemic [6,21], people might have intergenerational telephone and virtual contact, and such contact might play a role on ageism and attitude toward the COVID-19 vaccination passport.

The current research has two major goals. First, we aimed at shedding light on intergenerational contact during the COVID-19 pandemic, by exploring whether direct intergenerational contact decreased during the pandemic, and by analyzing both direct intergenerational contact (before and during the pandemic) and telephone and virtual intergenerational contact as predictors of ageism. Second, we aimed at analyzing whether intergenerational contact and ageism are associated with attitude toward the COVID-19 vaccination passport. In the following paragraphs, we review and summarize the literature on intergenerational contact during the COVID-19 pandemic and the literature supporting the hypothesized associations between intergenerational contact, ageism, and attitude toward the COVID-19 vaccination passport. 

### 1.1. Intergenerational Contact during the COVID-19 Pandemic

Intergroup contact has been consistently found to be one of the main strategies to reduce prejudice [22,23], especially when contact is perceived and experienced as positive [24,25]. Specifically, knowledge of one or more members of an external group (i.e., outgroup), especially if interactions with outgroup members are frequent and positive, has been found to reduce prejudice against the whole outgroup [26]. Applying intergroup contact theory to age relationships, intergenerational contact has been found to be generally perceived and experienced as positive and pleasant [27] and to be associated with reduced ageism [17,18,19]. Research on intergenerational contact has usually distinguished between contact quantity and contact quality, finding that contact quality has stronger effects on ageism reduction compared to contact quantity [15,28,29].

While the effectiveness of intergenerational contact for ageism reduction is well established, the COVID-19 pandemic has dramatically changed the frequency and the nature of intergenerational contact. Indeed, people have been called to separate and to avoid face-to-face interactions to reduce the spread of coronavirus. The mass media and political communication have underlined the vulnerability of older people to the COVID-19 [6], and thus direct, face-to-face intergenerational contact might have been avoided [20]. The COVID-19 pandemic has also been characterized by calls for intergenerational solidarity, and for finding alternative contact forms which might prevent loneliness among older people [12]. Therefore, alternative virtual (e.g., via telephone or different online tools) contact might have increased and played a role in intergenerational relations and ageism.

Research reports mixed findings about the nature and changes of intergenerational contact during the COVID-19 pandemic. McDarby, Ju, and Carpenter [30] assessed different forms of contact (via telephone and online platforms) between American young adults and their grandparents (April 2020) and found that telephone and online contact increased during the pandemic compared to before the pandemic, mainly because of worry about grandparents. Vergauwen et al. [31] examined a large cross-country dataset (June–July 2020) on older adults’ contact with non-cohabitating children and found that overall contact did not decrease during the pandemic, but rather increased for a large proportion of respondents and decreased only for a minority of respondents. Similarly, Podhorecka et al. [32] found that only a minority of Polish respondents diminished or avoided contact with older people (data collected in February 2021). However, Vergauwen et al. [31] and Podhorecka et al. [32] did not distinguish between direct vs. telephone and online contact in their analysis. Instead, Arpino, Pasqualini, and Bordone [20] distinguished between physical (direct, face-to-face) and non-physical (e.g., video-calls, instant messaging) contact, and analyzed older people’s intergenerational contact during the first lockdown in Italy, Spain, and France (April 2020). They found that overall physical intergenerational contact diminished, while non-physical intergenerational contact increased compared to before the pandemic. Using the same dataset, Arpino, Pasqualini, Bordone, and Solé-Aurò [33] further demonstrated the importance of non-physical contact which was associated with reduced risk of depression among older people.

Overall, research on intergenerational contact during the COVID-19 pandemic suggests that physical, face-to-face contact has diminished, while non-physical (e.g., telephone, virtual) contact has increased, and that such non-physical contact could contribute to well-being of older adults. While this research sheds light on several facets of intergenerational contact during the pandemic, additional issues need to be explored. First, previous research has not tested associations between different intergenerational contact forms (physical vs. non-physical, before or during the pandemic) and ageism. Second, published research was conducted mainly in the first months of the pandemic, for an exception see [32]. While that phase is very intriguing for research on intergenerational contact because of the very strong call to separate and to avoid physical contact especially with older people, contact habits can change, and therefore intergenerational contact should also be investigated during subsequent phases of the pandemic. Third, most of the research has examined only the point of view of older adults, while the point of view of non-older adults has remained underexplored, for exceptions see [30,32].

### 1.2. Intergenerational Contact, Ageism, and Attitudes and Behaviors during the COVID-19 Pandemic

Vulnerability of older people to COVID-19 has been established by epidemiological research, showing higher mortality rates and higher rates of serious complications following COVID-19 infection among older people [34]. Such vulnerability has been widely stressed by the mass media and in political discourse, with calls to protect older people from COVID-19 [6].

The pandemic is therefore considered as an intergenerational issue [5], and ageism might play a role in attitudes and behaviors related to the pandemic. If certain behaviors (e.g., preventive behaviors such as wearing face masks, using sanitizing gel) are aimed at reducing the spread of coronavirus which is particularly dangerous for older people, then ageism might be negatively associated with the implementation of such preventive behaviors. Indeed, research has analyzed whether attitude toward older people and different forms of ageism underlie attitudes (e.g., toward use of limited healthcare resources) and behaviors (e.g., preventive behaviors aimed at reducing the spread of coronavirus) during the pandemic. 

For example, Graf and Carney [13] analyzed associations between hostile ageism, benevolent ageism, and intentions of social distancing, and found that both hostile and, unexpectedly, also benevolent ageism were associated with reduced intentions of physical distancing. Similarly, Vale et al. [14] ran an online survey with young and old American participants reporting their hostile and benevolent ageism, pandemic-related fear, behavioral change to prevent the spread of coronavirus, and support for physical distancing. They found that hostile ageism and, unexpectedly, also benevolent ageism were negatively associated with behavioral change and support for physical distancing. However, benevolent ageism was indirectly associated with more behavioral change and support for physical distancing via increased pandemic-related fear. Visintin [15], in turn, analyzed associations between different forms of ageism and two forms of preventive behaviors (i.e., maintaining physical distance and using protection devices such as face masks and sanitizing gel) among non-older people during the first lockdown in Italy, and found that both favorable attitude toward older people and benevolent ageism were associated with the more frequent use of protection devices, while only favorable attitude toward older people was associated with physical distancing.

Turning to prosocial behaviors, Lyte et al. [35] provided evidence that ageism among American undergraduates assessed before the COVID-19 pandemic (Fall 2019) negatively predicted prosocial behavior during the pandemic (Spring 2020) such as helping or contacting vulnerable people, while the perception of older people as incompetent assessed before the pandemic predicted more prosocial behavior toward vulnerable people during the pandemic. Apriceno et al. [16], in turn, found that, among American undergraduates, hostile ageism was associated with lower ratings of prioritizing older people in getting COVID-19 triage, testing, and vaccine, while benevolent ageism was positively associated with higher priority ratings. Focusing on intergenerational tensions, Spaccatini et al. [36] ran an online survey among young Italian respondents assessing ageism, attributions of culpability for the severity of COVID-19 restrictions to older people, and attitudes toward the selective isolation of older (but not young) people and selective lockdown of older people. They found that ageism was positively associated with attributions of culpability to older people for the severity of COVID-19 restrictions, and consequently with support for isolation and lockdown for older people only.

To summarize, previous research analyzing associations between ageism and attitudes and behaviors during the pandemic has consistently found undesirable effects of unfavorable attitude toward older people and hostile ageism which were associated with lower preventive behaviors [13,14,15], reduced helping behaviors [30], and beliefs that older people should not be prioritized in COVID-19 treatment [16] or should be isolated during the pandemic [36]. The effects of benevolent ageism on attitudes and behaviors during the pandemic are instead less consistent [13,14,15,16,35]. Here we extended previous research by analyzing an uninvestigated possible outcome of ageism during the pandemic, i.e., attitude toward the COVID-19 vaccination passport. Such a policy aimed at limiting the spread of coronavirus and preserving people’s health, because vaccinated people are less likely to get infected and to transmit coronavirus [2,3]. Therefore, the COVID-19 vaccination passport policy might be perceived as a policy aimed at preserving the health of vulnerable or older people, and ageism might be negatively associated with support for such policy.

Some research also analyzed intergenerational contact as predictor of attitudes and behaviors during the COVID-19 pandemic. In the previously described study, Visintin [15] showed that quality of intergenerational contact before the pandemic was associated with more preventive behaviors, partly via more favorable attitude toward older people. Podhorecka et al. [32], in turn, analyzed several facets of Polish respondents’ contact with older people (e.g., importance attributed to contact with older people, intergenerational contact across different contexts such as work and family), and found that the importance attributed to contact with older people was associated with positive attitude toward prioritizing older people in COVID-19 treatment and vaccination, and with positive attitude toward governmental measures to preserve older people’s health during the pandemic (i.e., implementing designated shopping hours for older people). Therefore, in this research we tested whether intergenerational contact was associated with positive attitude toward the COVID-19 vaccination passport.

### 1.3. Overview of the Current Research

We ran a survey with Italian respondents in November 2021. Italy has been one of the countries most hit by the pandemic (https://www.worldometers.info/coronavirus/; accessed on 22 August 2022). The COVID-19 vaccination campaign in Italy started on 27 December 2020, and initially targeted healthcare professionals and older and physically vulnerable people, to subsequently reach all the population. Italy implemented strict policies aimed at reducing the spread of coronavirus. Crucially for the current research, the COVID-19 vaccination passport (in Italy labelled “green pass”) policy forced people who were not vaccinated or did not previously have COVID-19 to get tested against COVID-19 to be able to enter workplaces, bars and restaurants, indoor recreational activities, schools and universities, with the validity of the test lasting 2 days. Moreover, in a specific time (from 15 February 2022 until 31 March 2022 for workplaces; from 15 February 2022 until 30 April 2022 for indoor recreational activities), entrance was allowed only with a full vaccination cycle or with certificate of recovery from COVID-19 (i.e., with the so-called “green pass rafforzato”).

In a correlational study, we investigated intergenerational contact dynamics during the COVID-19 pandemic, and associations between intergenerational contact, ageism, and attitude toward the COVID-19 vaccination passport. While also older people can have ageist stereotypes and attitudes [37,38], here we focused on the point of view of non-older people and their contact with and attitudes toward older people.

Specifically, we aimed at shedding light on quantity and quality of (direct) intergenerational contact before and during the COVID-19 pandemic. Following calls to physically distance and to especially reduce intergenerational contact because of the vulnerability of older people, we expected direct contact frequency to decrease during the pandemic compared to before the pandemic [20]. Telephone and virtual contact might instead be relatively frequent, in line with calls for intergenerational solidarity [6,21] and with previous research [20,30]. Turning to contact quality, we expected intergenerational contact to be perceived as positive and pleasant in line with previous research [27], but we explored possible differences in contact quality between the three intergenerational contact forms under investigation. 

Previous research has established negative associations between intergenerational contact and ageism [17,18,19], with stronger effects of contact quality compared to quantity, the latter often not significantly associated with ageism [15,28,29]. We expected to replicate the negative associations between intergenerational contact quality and ageism, and further explored which intergenerational contact forms under investigation were significantly associated with ageism when controlling for the other contact forms, to provide information about which contact facets are more consequential for ageism.

Finally, we examined intergenerational contact and ageism as predictors of attitude toward the COVID-19 vaccination passport. When predicting attitude toward the COVID-19 vaccination passport, we controlled for attitude toward COVID-19 vaccination. Indeed, individuals’ vaccination attitudes and choices are likely to reflect the willingness to protect their own health and the health of the people one meets face-to-face, and thus pertain mainly to private health concerns. Instead, attitude toward the vaccination passport is likely to reflect a broader desire for people to be vaccinated in order to reduce the spread of the coronavirus and protect vulnerable people, and therefore pertain mainly to public health concerns, see [39]. Given the possible high correlation between the two attitudes, and with the aim of isolating the effects on attitude toward the vaccination passport which mainly pertains to public health, we therefore controlled for attitude toward COVID-19 vaccination. Based on the literature showing negative associations between unfavorable, hostile attitude toward older people and attitudes and behaviors aimed at reducing the spread of coronavirus [13,14,15,16], we hypothesized a negative association between ageism and attitude toward the COVID-19 vaccination passport. Based on the literature showing that intergenerational contact is associated with reduced ageism, and on the findings by Visintin [15] that intergenerational contact quality was associated with more preventive behaviors via reduced ageism, we tested indirect effects between intergenerational contact and attitude toward the COVID-19 vaccination passport via ageism.

## 2. Materials and Methods

### 2.1. Participants and Procedure

Participants were 153 university students from an Italian university (52% Bachelor students and 48% Master students). They were recruited from students attending a Bachelor or a Master course in social psychology during the fall 2021 semester. All students attending the courses were invited to respond to a Google form online questionnaire about the COVID-19 pandemic. For participation respondents received partial course credit. All participants provided full informed consent. Among the 153 students who answered to the questionnaire, 78% were female (1 missing data). 97% of participants had Italian nationality and no participant declared living outside Italy at the time of data collection (3 missing data). Mean age was 25.80 (*SD* = 7.19; range = 20–51). 

### 2.2. Measures in the Questionnaire

*Intergenerational contact.* A battery of items investigated intergenerational contact, distinguishing between three intergenerational contact forms: direct contact before the COVID-19 pandemic, direct contact during the COVID-19 pandemic, and telephone and virtual (e.g., via WhatsApp, Telegram, Facebook, Skype) contact. For each contact form we distinguished between contact quantity and contact quality (measures adapted from [15,40]). Contact quantity was assessed with a single question inviting respondents to assess the frequency of their contact with older people on a response scale from 1 (*very rarely*) to 5 (*very often*). Contact quality was assessed with three items inviting respondents to evaluate such contact from *unpleasant* (1) to *pleasant* (5), from *involuntary* (1) to *voluntary* (5), and from *negative* (1) to *positive* (5). Contact quality measures were reliable (Cronbach alphas between 0.84 and 0.89), and answers were averaged to create composite scores with higher values representing higher contact quality.

*Ageism. Ageism* was assessed with a single item asking respondents to report their attitude toward older people (0 = *extremely unfavorable*; 10 = *extremely favorable*). Answers were reverse coded so that higher values represent higher ageism. 

*Attitude toward the COVID-19 vaccination passport.* Respondents were asked to report their attitude toward the COVID-19 vaccination passport, on a scale ranging from 0 (*extremely unfavorable*) to 10 (*extremely favorable*). 

*Attitude toward COVID-19 vaccination*. Respondents were asked to report their attitude toward COVID-19 vaccination on two items (adapted from [41]) from 0 (*extremely unfavorable*) to 10 (*extremely favorable*) and from 0 (*extremely doubtful*) to 10 (*not at all doubtful*). Answers were averaged to create a reliable composite score (Spearman Brown reliability = 0.82) with higher scores representing better attitude toward the COVID-19 vaccination. 

*Previous experiences with COVID-19*. Respondents also answered questions about their previous experiences with COVID-19. Specifically, they were asked whether they had COVID-19, how many people they knew who had COVID-19 on a scale from 0 (*none*) to 5 (*more than 20*), and whether they lost loved ones because of COVID-19. 

The questionnaire included additional measures. Details about such measures can be obtained upon request from the corresponding author.

## 3. Results

### 3.1. Preliminary Data Analysis

Missing data were 0.003% and were missing completely at random (Little MCAR test, χ^2^(80) = 80.08, *p* = 0.476), and were therefore imputed using the EM algorithm in SPSS. 

Descriptive statistics and bi-variate correlations are reported in Table 1. As shown in Table 1, respondents exhibited low ageism, while attitudes toward the COVID-19 vaccination passport and toward the COVID-19 vaccination were rather positive. Regarding participants’ previous experiences with COVID-19 (descriptive statistics not displayed in Table 1), 11 participants had COVID-19 (2 missing data), while 17 participants reported the loss of loved ones because of COVID-19. Finally, mean score of how people who had COVID-19 one knows was 1.80 (*SD* = 1.23), close to response option 2 (*5 to 10 people*).

### 3.2. Intergenerational Contact and Ageism

First, we ran ANOVAs to test whether contact quantity and quality vary as a function of the form of intergenerational contact (direct before the pandemic vs. direct during the pandemic vs. virtual). We found an effect of contact form on contact quantity, *F*(1.91, 290.11) = 39.41, *p* < 0.001. As hypothesized, direct contact during the pandemic was less frequent than direct contact before the pandemic (*p* < 0.001). Contrary to the hypothesis, virtual contact was less frequent than both forms of direct contact (*p* < 0.007) (see Table 1). 

We also found an effect of contact form on contact quality, *F*(2, 304) = 11.89, *p* < 0.001. While all contact quality scores suggest high quality intergenerational contact (see Table 1), quality of direct contact before the pandemic was higher than quality of direct contact during the pandemic and than quality of virtual contact (*p* < 0.001), with no significant difference between the scores of quality of direct contact during the pandemic and of quality of virtual contact (*p* = 0.316). 

Next, we examined the different contact facets as predictors of ageism. Bi-variate correlations suggested that, for all contact forms, contact quality correlated negatively with ageism, whereas contact quantity negatively correlated with ageism only for direct contact before the pandemic and virtual contact (Table 1). To examine the effect of each contact facet on ageism over and above the effects of other contact facets, we ran a multiple regression analysis with quantity and quality of direct contact before the pandemic, quantity and quality of direct contact during the pandemic, and quantity and quality of virtual contact simultaneously entered as predictors of ageism. Gender and age were included as control variables (Table 2). Only quality of direct contact before the pandemic was significantly associated with reduced ageism, suggesting this contact facet as the most consequential for ageism. 

### 3.3. Intergenerational Contact, Ageism, and Attitude toward the COVID-19 Vaccination Passport

We examined ageism as a predictor of attitude toward the COVID-19 vaccination passport. To isolate the effect of attitude toward the COVID-19 vaccination passport from attitude toward the COVID-19 vaccination, in the multiple regression analysis we controlled for attitude toward the COVID-19 vaccination. Additional control variables were gender, age, having had COVID-19, loss of loved ones because of COVID-19, and number of people who had COVID-19 one knows (Table 3). As hypothesized, we found that ageism was negatively associated with attitude toward the vaccination passport. 

**Figure 1:**
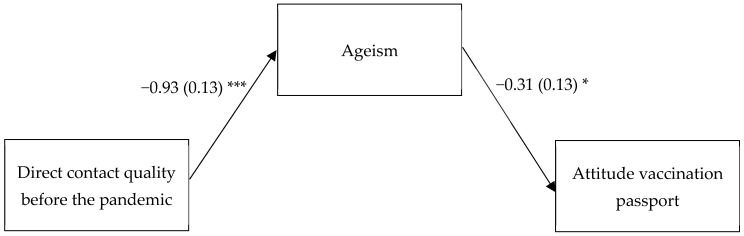
Indirect effect of quality of direct intergenerational contact before the pandemic on attitudes toward the COVID-19 vaccination passport via ageism. Control variables: gender, age, having had COVID-19, loss of loved ones because of COVID-19, number of people who had COVID-19 one knows, and attitudes toward the COVID-19 vaccination. The direct association between intergenerational contact and attitudes toward the COVID-19 vaccination passport was not significant (*B* = −0.03, *SE* = 0.23, *p* = 0.905). *** *p* < 0.001. * *p* < 0.05.

Finally, we examined indirect effects from intergenerational contact (quality) on attitude toward COVID-19 vaccination passport via ageism. We focused on quality of direct contact before the pandemic which was the only significant predictor of ageism in the regression analysis (see Table 2). In the multiple regression analysis, we controlled for gender, age, having had COVID-19, loss of loved ones because of COVID-19, number of people who had COVID-19 one knows, and attitude toward the COVID-19 vaccination (Figure 1). We found an indirect effect of quality of direct contact before the pandemic on attitude toward the COVID-19 vaccination passport via ageism, *B* = 0.29, *SE*_(boot)_ = 0.16, 95% CI = [0.05,0.68]. Results did not change when other intergenerational contact forms were controlled for. 

## 4. Discussion

We conducted a correlational study during the fourth wave of the COVID-19 pandemic in Italy analyzing non-older people’s intergenerational contact, ageism, and attitude toward the COVID-19 vaccination passport. At the time of data collection, the vaccination passport policy was ongoing in Italy, therefore people needed to be vaccinated or to have previously had COVID-19 or to have a recent negative COVID-19 test to enter workplaces and several public spaces such as bars and restaurants, schools and universities. Restrictions increased during the months following data collection, with the introduction of “green pass rafforzato” (i.e., a vaccination passport based only on vaccination or previous positivity to COVID-19 test) and the vaccination obligation for people over 50 and for people working in healthcare, education, and law enforcement sectors. Such measures elicited harsh protests and criticism [1]. It is therefore important to investigate correlates of support vs. opposition to such policies. As the pandemic has also been framed by the mass media and political discourse as an intergenerational issue [5,6], we investigated intergenerational contact and ageism as possible correlates of attitude toward the vaccination passport. 

Our findings corroborate the literature on associations between ageism and attitudes and behaviors during the pandemic. Specifically, previous research showed that ageism was associated with less preventive behaviors to reduce the spread of coronavirus [13,14,15], and with worse attitudes toward physical distancing [14] and toward prioritizing older people in COVID-19 treatment [16]. We extend such literature by providing evidence that ageism is also associated with negative attitude toward the vaccination passport. Given that the vulnerability of older people to coronavirus has been stressed in the mass media and political discourse, and that the vaccination passport policy might contribute to reducing the spread of coronavirus and preserving health of older people, negative attitude toward older people underlie negative attitude toward such a policy.

Furthermore, our research sheds light on intergenerational contact dynamics during the pandemic. While previous research on intergenerational contact mostly focused on the first wave of the pandemic [15,20,30,31], when there were strong restrictions to face-to-face interactions with other people, it is important to also analyze intergenerational contact in other phases of the pandemic. Indeed, intergroup contact habits might change over time, and reduction in intergenerational contact in a specific time point might have long lasting effects. Furthermore, even when restrictions to face-to-face contact are released and despite vaccinations, the COVID-19 pandemic is not over, and people are still getting infected by coronavirus. In our view, it is therefore possible that people might be reluctant to meet older people who might still be perceived as vulnerable to coronavirus. Our findings corroborate the literature from the first wave of the pandemic [20] suggesting that direct intergenerational contact decreased during the pandemic. People had some telephone and virtual contact with older people, but such telephone and virtual contact was not frequent compared to direct contact, suggesting a possible digital divide [21]. Turning to contact quality, in line with previous research [27] all the investigated forms of contact with older people were perceived as positive. However, both direct contact during the pandemic and telephone and virtual contact were perceived as less positive than direct contact before the pandemic. We speculate that contagion fear and anxiety played a role in direct intergenerational contact during the pandemic, reducing perceived quality compared to direct intergenerational contact before the pandemic. Future research should test this possibility. Turning to telephone and virtual intergenerational contact, this might be perceived as less positive than direct contact before the pandemic because it might be experienced as less enriching and deep than direct contact, or again because of digital barriers which prevent fully pleasant intergenerational virtual exchanges. These explanations should be tested by future research. Quality of direct contact before the pandemic was also the most consequential contact facet, being associated with ageism when other contact facets were controlled for. Overall, our findings provide a somehow pessimistic view of changes in intergenerational dynamics during the pandemic, because quality of the most consequential contact form (i.e., direct contact) diminished during the pandemic. While telephone and virtual contact could help to reduce loneliness and depression [33], it was less frequent and less consequential for ageism than direct contact. 

It is noteworthy that some research questioned associations between intergenerational contact and the diffusion of coronavirus and severity of COVID-19 disease [42,43]. While discussion of such findings goes beyond the scope of the current article, we hope that the quality of intergenerational contact will soon further improve to at least the pre-pandemic level, given its importance for well-being and for intergenerational attitudes and discrimination.

Our study also contributes to the literature on the intergroup contact tertiary effect, i.e., intergroup contact effects beyond intergroup relations [44,45]. Indeed, previous research has shown that intergroup contact can be associated for example with more pro-environmental behavior [46] or with organizational outcomes such as work engagement and reduced turnover intentions [47]. In this research we show that intergroup contact effects might also pertain to public health, being (indirectly via ageism) associated with attitudes toward the vaccination passport, see also [15].

Despite these contributions, some limitations of the current research should be acknowledged. First, our sample was relatively small and non-representative, including only university students who are by definition highly educated, who usually exhibit low prejudice and, in this study, overall positive attitude toward the vaccination passport, and who might have specific living conditions which affect their intergenerational contact (e.g., living far away from grandparents to attend university). Future research should use larger representative samples to grasp the variety of intergenerational contact experiences and of attitudes toward older people and toward the vaccination passport and other pandemic-related policies. Second, data were correlational, and causality could not be established. Future research should replicate and extend our findings with longitudinal or experimental research designs. For example, longitudinal research could clarify intergenerational contact changes over time. Experimental research could provide insights into whether mentioning the protection of older people in campaigns aimed at increasing preventive behaviors and vaccinations could contribute to the efficacy of such campaigns. Third, while we distinguished between direct intergenerational contact before the pandemic and direct intergenerational contact during the pandemic, we did not distinguish between telephone and virtual intergenerational contact before and during the pandemic, and therefore we cannot know whether such contact increased during the pandemic. Future research should distinguish such contact facets and investigate whether telephone and virtual intergenerational contact increased during the pandemic. Finally, it is worth mentioning that our findings might be specific to the country under investigation (Italy) which has a large share of older adults, and which has implemented strict COVID-19 policies. Future studies should replicate and extend our findings in other countries.

## 5. Conclusions

Despite these limitations, this research suggests intergenerational contact and ageism as factors contributing to public health attitudes. We believe positive, cooperative, and egalitarian intergenerational relationships can contribute not only to reduce ageism but also to the well-being and health of people of any age. Therefore, we encourage the mass media and governments to keep in mind the importance of intergenerational relationships when designing campaigns and policies aimed at preserving public health.

## Figures and Tables

**Table 1:** Means, standard deviations, and correlations between variables.

	Mean (SD)	1	2	3	4	5	6	7	8
1. Quantity DIC BP	3.23 (1.24)	-							
2. Quality DIC BP	4.15 (0.82)	0.53 ***	-						
3. Quantity DIC DP	2.58 (1.18)	0.42 ***	0.39 ***	-					
4. Quality DIC DP	3.96 (0.92)	0.37 ***	0.76 ***	0.43 ***	-				
5. Quantity TVIC	2.33 (1.16)	0.31 ***	0.35 ***	0.51 ***	0.35 ***	-			
6. Quality TVIC	3.91 (0.88)	0.28 ***	0.69 ***	0.31 ***	0.76 ***	0.42 ***	-		
7. Ageism	1.44 (1.51)	−0.25 **	−0.53 ***	−0.14	−0.43 ***	−0.20 *	−0.46 ***	-	
8. Attitude VP	7.67 (2.87)	−0.001	−0.03	−0.12	−0.03	−0.02	−0.04	−0.12	-
9. Attitude V	7.89 (2.40)	0.04	−0.13	−0.17 *	−0.08	−0.18 *	−0.09	0.03	0.73

Notes: *** *p* < 0.001. ** *p* < 0.001. * *p* < 0.05. DIC = Direct Intergenerational Contact. BP = Before the Pandemic. DP = During the Pandemic. TVIC = Telephone and Virtual Intergenerational contact. VP = COVID-19 Vaccination Passport. V = COVID-19 Vaccination.

**Table 2:** Multiple regression analysis predicting ageism.

	Ageism
Intercept	6.34 (0.62) ***
Quantity DIC BP	−0.04 (0.10)
Quality DIC BP	−0.69 (0.22) **
Quantity DIC DP	0.14 (0.11)
Quality DIC DP	−0.09 (0.20)
Quantity TVIC	0.02 (0.11)
Quality TVIC	−0.29 (0.20)
Age	−0.01 (0.01)
Gender	−0.73 (0.25) **
*R^2^*	0.35
F	9.47 ***
*df*	8, 144

Notes: Unstandardized coefficients (and standard errors) are reported. *** *p* < 0.001. ** *p* < 0.01. DIC = Direct Intergenerational Contact. BP = Before the Pandemic. DP = During the Pandemic. TVIC = Telephone and Virtual Intergenerational contact. For gender, 0 = male and 1 = female.

**Table 3:** Multiple regression analysis predicting attitudes toward the COVID-19 vaccination passport.

	Attitudes Vaccination Passport
Intercept	0.68 (1.07)
Ageism	−0.30 (0.11) **
Attitudes COVID-19 vaccination	0.89 (0.07) ***
Age	0.01 (0.02)
Gender	−0.32 (0.40)
Had COVID-19	0.20 (0.61)
Loss because of COVID-19	−0.66 (0.51)
People who had COVID-19 one knows	0.19 (0.13)
*R* ^2^	0.57
F	28.00 ***
*df*	7, 145

Notes: Unstandardized coefficients (and standard errors) are reported. *** *p* < 0.001. ** *p* < 0.01. For gender 0 = male and 1 = female. For Had COVID-19 and Loss because of COVID-19, 0 = No and 1 = Yes.

## Data Availability

Data can be retrieved at: https://doi.org/10.6084/m9.figshare.20198669 (accessed on 23 August 2022).
